# From Aggregation to Dispersion: How Habitat Fragmentation Prevents the Emergence of Consensual Decision Making in a Group

**DOI:** 10.1371/journal.pone.0078951

**Published:** 2013-11-11

**Authors:** Grégory Sempo, Stéphane Canonge, Jean-Louis Deneubourg

**Affiliations:** Unit of Social Ecology, Université libre de Bruxelles, Brussels, Belgium; University of Maribor, Slovenia

## Abstract

In fragmented landscape, individuals have to cope with the fragmentation level in order to aggregate in the same patch and take advantage of group-living. Aggregation results from responses to environmental heterogeneities and/or positive influence of the presence of congeners. In this context, the fragmentation of resting sites highlights how individuals make a compromise between two individual preferences: (1) being aggregated with conspecifics and (2) having access to these resting sites. As in previous studies, when the carrying capacity of available resting sites is large enough to contain the entire group, a single aggregation site is collectively selected. In this study, we have uncoupled fragmentation and habitat loss: the population size and total surface of the resting sites are maintained at a constant value, an increase in fragmentation implies a decrease in the carrying capacity of each shelter. For our model organism, *Blattella germanica*, our experimental and theoretical approach shows that, for low fragmentation level, a single resting site is collectively selected. However, for higher level of fragmentation, individuals are randomly distributed between fragments and the total sheltered population decreases. In the latter case, social amplification process is not activated and consequently, consensual decision making cannot emerge and the distribution of individuals among sites is only driven by their individual propensity to find a site. This intimate relation between aggregation pattern and landscape patchiness described in our theoretical model is generic for several gregarious species. We expect that any group-living species showing the same structure of interactions should present the same type of dispersion-aggregation response to fragmentation regardless of their level of social complexity.

## Introduction

Living in groups is most likely the most common collective behavior among organisms, in vertebrates, invertebrates and unicellular organisms [1]–[6]. The presence of this aggregative behavior at all living scales is positively related to the fitness of the group [7]. Indeed, the benefits of group living are numerous [2]: reduction in predation risk by dilution or confusion [8], facilitation of foraging [9], and water loss regulation and thermoregulation [10], [11]. These benefits reflect different underlying Allee effects [12], [13] and are largely dependent on environmental characteristics. Limited resources and/or an increase in the population density will inevitably lead to competition between individuals for access to resources, reducing *ipso facto* the benefits of group living. Thus, at any given time, the size of a group results from a balance between benefits and costs, such as the sharing of food resources [14], [15], intensification of competition between sex partners [16], and increased epidemic risks [17].

The adaptive value of group living has been widely discussed, yet the number of studies devoted to investigating the proximal causes and behavioral mechanisms governing the emergence of various spatio-temporal distributions of organisms remains small. Pioneer studies in this area have identified two processes involved in the formation of an aggregate: individual behavioral modulations due to heterogeneities of the environment on the one hand [18] and social interactions on the other hand [2], [4], [6]. In the second case, the aggregation results from a social amplification of a signal or a cue emitted by conspecifics and is a by-product that emerges from the local interactions between individuals, without personal knowledge of the global group distribution (ant [19]; cockroach [20]–[23]). These two processes may act together: aggregate formation at a given site could be based on a double modulation of the individual probability of leaving the aggregate depending on the abiotic or biotic characteristics of the site and the presence of conspecifics [24], [25]. Although the factors leading to aggregation or dispersion have previously been discussed in the literature, little work has been devoted to the dispersion-aggregation transition and synergy between the various factors governing group structure.

For gregarious species for which cooperativity between individuals plays a particularly important role, optimality is reached when the individuals come together at the same site [20]. Indeed, the choice of habitat is a crucial life history trait for individuals, as it provides protection against physical assault related to the environment and predation and affects most of the components of reproductive success [26]. Each individual attempts to select the most favorable shelter. However, the habitat quality may depend on many external factors, such as the presence of predators [27], food availability [28], and the type of vegetation [29]. When the number of parameters is large, the value assessment of a site can be more costly in energy and time. The most parsimonious and most effective selection strategy is, therefore, based on the use of indices integrating the effects of different environmental factors [30], [31]. For social species, public information [32] related to the presence of conspecifics is an indication of habitat quality during the selection process of the best site [33]–[38]. However, the resulting increase in density can lead to suboptimal solutions linked to an increase of competition between individuals. Habitat fragmentation, either produced naturally or by anthropogenic modification, also exerts a strong influence on the distribution of individuals [39] and may alter the interactions between species [40]. Defined as a division of the living area, “fragmentation” is distinguished from “habitat loss”, which corresponds to the reduction of its surface area [41]. Although the negative influence of habitat loss is well documented, the negative contribution of fragmentation is still under debate [41]–[43]. Fragmentation of the total habitat area has the dual effect of increasing the number of habitable sites, corresponding to the fragments, and of reducing their individual area. In such an environment, the coupling of (1) the “maintenance of social cohesion” (i.e., being in the presence of the majority of the population at the same site), (2) the fact of “being as soon as possible under the protection of a shelter”, and (3) the competition between group members seems hard to achieve, requiring compromise among the individuals.

Within this context, the objective of this study is to highlight the behavioral mechanisms that govern the decision-making process leading to the selection of a resting site in a fragmented environment using our gregarious model species, the cockroach *Blattella germanica* (L.). While maintaining the same shelter quality, we sought to highlight and understand how a group of cockroaches respond to the presence of a variable number of shelters, with an increase being correlated to a reduction of the carrying capacity of each of shelter. The reduction of the carrying capacity is used to evaluate specifically the contribution of the presence of congeners in the spatio-temporal organization of individuals. The classical ideal free distribution theory [44] assumes that individuals faced with such fragmentation are distributed homogeneously among sites to maximize their individual gain. As this theory considers individuals as competitors, more of them are present on a site less will be the profitability of this site. However, according to the theoretical model developed by recent studies on gregarious species [20], [21], [45], [46], the distribution of individuals instead corresponds to the selection of a minimum number of shelters. This prediction relies on an amplification process via a positive feedback. When the population size at the shelter increases, the individual probability of leaving it decreases and, consequently, newcomers are “trapped” in the shelter. This process is a nonlinear phenomenon that depends on the population density at a given site and highlights the existence of a quorum, corresponding to the number of individuals that is necessary and critical for the nucleation of an aggregate [45].

In this study, we tested, at the collective level, the influence of the fragmentation level without habitat loss on the spatio-temporal distribution of a gregarious species. Our experimental device, which is based on the system “cockroaches-shelter” [21], [23], [47], facilitates modifications of the extent of fragmentation. This particular organization implies that individuals forage alone during the night and aggregate during daylight hours. As the environment comprises several potential resting sites, the individuals have to make a consensual decision to achieve the group size that maximizes the positive Allee effects [12]. Indeed, several studies have shown that cluster formation provides several advantages to each individual cockroach. Notably, it was demonstrated that clustering helps to maintain the humidity [11] and temperature conditions. This consensus emerges from the local interactions between individuals and the resulting amplifications.

Based on two complementary approaches, we test the influence of different fragmentation level on cockroaches’ behaviors. The first addresses the temporal dimension, i.e., the analysis of the temporal evolution of the total number of cockroaches sheltered, whereas the second focuses on the spatial dimension, i.e., the analysis of the spatial distribution of cockroaches in the shelters. By considering the complex network of negative (e.g., crowding) and positive (e.g., peer number) feedback involved in aggregation processes, our double approach will take a first step toward a better understanding of the influence of fragmentation on the spatio-temporal organization of gregarious species and the mechanisms involved.

## Methods

### Breeding Conditions

The cockroaches tested correspond to a population bred in the Service d’Ecologie Sociale (ULB, Belgium) since 2004. Individuals of both sexes and of all larval instars were housed in 12 transparent plastic boxes (34×24×14 cm). Cockroaches have folded cardboard and paper shelters at their disposal. Food (dog food pellets, Tom & Co ®) and water were provided *ad libitum*.

The breeding room was maintained at 22±2°C, with 40±10% RH and a photoperiod of 12∶12 h L:D.

### Experimental Setup

The experimental setup consisted of a circular arena (height: 4.5 cm, diameter: 24 cm) coated with fluon to prevent the cockroaches from escaping and to confine them in a 2-D space. The ground of the experimental arena was covered with a sheet of white paper (120 g/m2). Illumination was provided by a lamp bulb centered on the experimental arena (20 W; Philips Ambiance Pro, Philips Belgium NV, Brussels, Belgium), providing 1155 lux at the ground level.

According to the level of fragmentation of the environment, 1 (F1), 2 (F2), 6 (F6), or 10 (F10) shelters consisting of Plexiglas discs were placed symmetrically on a circle of 21 cm centered in the arena. These shelters were maintained at 5 mm above the ground level by three needles affixed to the periphery. As the total shelter surface was maintained constant at 25.13 cm^2^ (5.6% of the arena surface), the individual surface area for F1, F2, F6, and F10 was 25.13, 12.56, 4.19, and 2.51 cm^2^, respectively (Table 1). Based on our observations, the total carrying capacity for 25.13 cm^2^ of shelter is approximately 30 individuals, exceeding the tested population size (16 cockroaches). Indeed, by taking into account only conditions under which shelter size implies crowding, the carrying capacity of a shelter is strongly correlated to its surface area, with a maximum number of individuals under a shelter observed in our experiments of 16, 7, and 5 for the F2, F6, and F10 shelters, respectively (Linear regression *r* = 0.99, *F_1,1_* = 4296, *p*<0.01, y = 1.09+2.35).

**Table 1:** Surface and diameter of the shelters for the 4 experimental conditions.

Experimental condition	Number of shelters	Surface of each shelter	Diameter
**F1**	1	25.13 cm^2^	5.56 cm
**F2**	2	12.56 cm^2^	4 cm
**F6**	6	4.19 cm^2^	2.3 cm
**F10**	10	2.51 cm^2^	1.8 cm

The luminosity under the shelters was decreased by covering shelters with two layers of red filters (75±5 lux; Rosco color filter, E-Color #019: Fire, Roscolab Ltd., London, UK). The choice of such a red-light shelter was driven by the two following observations: (1) cockroaches stop running as soon as they enter a shadowed area [48], and (2) cockroaches perceive an area illuminated by a red light as a shadow because of the lack of red light-sensitive photoreceptors in their compound eyes [49].

The entire setup was surrounded by an opaque white enclosure to prevent the cockroaches from perceiving visual landmarks outside the experimental arena. The temperature in the experimental setup was maintained at 22±2°C. The ground (white paper) was replaced between each experiment, and the shelters were cleaned with water and denatured alcohol (97.1% ethanol +2.9% ether).

Sixteen adult males were randomly selected from breeding boxes and introduced in the arena at 10 am for a total duration of 8 h. For conditions F1, F2, F6 and F10, we have performed 16, 21, 21 and 32 replications respectively.

#### Data analysis

Comparisons of densities (number of cockroaches/cm2) were performed using the One-Sample t-test [50]. The effect of time on the presence of individuals in shelters were tested using a multiple comparison of slopes and elevation test [50]. A Kruskal-Wallis analysis of variance by rank test, followed by a Dunn’s multiple comparisons post-hoc test if required [50], was used to determine the influence of the fragmentation level on the mean fraction of sheltered cockroaches and to compare our experimental and theoretical results. The comparison between theoretical and experimental distributions of individuals among the different shelters was tested using a Spearman Rank Correlation [50].

The significance of statistical tests was set at α = 0.05. The statistical analyses were performed using GraphPad Prism software. The numerical simulations were performed using MatLab.

## Results

### Sheltering Behavior

The shelters are well perceived as the preferred resting places in the arena. Indeed, the mean density values observed under all shelters for the 4 conditions (total sheltered cockroaches/total shelters surface) are all significantly different from that assumed in the case of a homogeneous distribution of individuals in the arena (homogeneous density: 0.035 cockroach/cm^2^. One sample *t*-test at *t* = 480 min. F1∶0.61±0.03 cockroach/cm^2^, *t_15_* = 81, *P*<0.0001. F2∶0.59±0.04 cockroach/cm^2^, *t_20_* = 75, *P*<0.0001. F6∶0.53±0.05 cockroach/cm^2^, *t_31_* = 53, *P*<0.0001. F10∶0.45±0.08 cockroach/cm^2^, *t_31_* = 28, *P*<0.0001).

**Figure 1:**
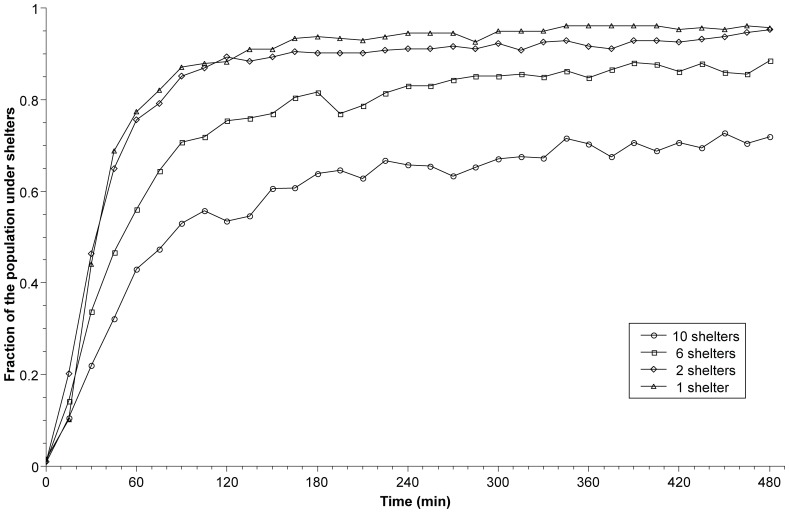
Dynamic of sheltering behavior. Change over time (min) of the mean fraction of cockroaches under shelters for groups confronted with a choice between 1 (n = 16, triangles), 2 (n = 21, diamonds), 6 (n = 21, squares), or 10 identical shelters (n = 32, circles).

For the 4 fragmentation levels tested, the fraction of the population aggregated inside all the shelters steeply increases during the first 60 minutes until reaching a plateau (Figure 1). However, the slopes of the fraction of the population observed during the first 60 minutes are different among the 4 tested conditions (*F_3,497_* = 26.5, *P*<0.0001). The comparison between slopes (*F_1,181_* = 0.65, *P* = 0.42) and intercepts (*F_1,182_* = 0.18, *P* = 0.67) of F1 and F2 indicate that they are not significantly different, with all other comparisons indicating a significant difference between the slopes. Therefore, between F2 and F10, the sheltering speed decreases with the fragmentation level (Figure 1).

After 480 minutes, when the population under the shelters is quite stable (Figure 1), the mean fraction of sheltered cockroaches for the 4 tested fragmentation levels is significantly different (Kruskal-Wallis test: *KW* = 59.1, *P*<0.0001). Indeed, we observe that the sheltered population decreases when the cockroaches are confronted with a choice between more than 2 shelters.

**Figure 2:**
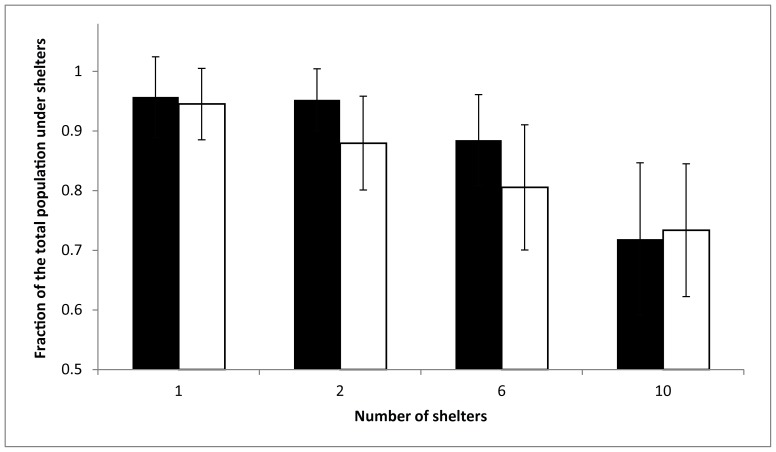
Theoretical and experimental influence of fragmentation on the sheltering behavior. Mean fraction of the total population under shelters (± S.D.) at t = 480 minutes for F1, F2, F6, and F10. Comparison of the experimental (black) and theoretical (white) data. Parameters values: μ = 0.0007, θ = 0.0049, n = 2.5, k = 4, ρ = 1296.

Based on the same decisions rules as those describing the decision-making process that allows *B. germanica* cockroaches to reach a consensus in a binary choice experiment [20], we are able to reproduce theoretically our experimental results, thus validating our hypothesis (Figure 2).

The evolution over time of the number of cockroaches *x_i_* under *p* shelters is as follows:
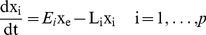
(1)where variable *x*_i_ represents the number of cockroaches present in shelter *i* and *x*_e_ is the number outside the shelters. The total number of individual (16 cockroaches) in the experimental setup is the sum of the individuals outside (x_e_) and inside the shelters:



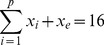
(2)The functions *E_i_* and *L_i_* denote the rate per individual of entering and leaving the shelter *i* and correspond to:
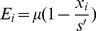
(3)

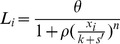
(4)


The function *E_i_* (Eq. 3) takes into account a crowding effect and decreases with the ratio between *x_i_*, the number of individuals present in shelter *i*, and its carrying capacity *s’*. The carrying capacity corresponds to the maximum number of cockroaches that can be hosted in one of the *p* shelters and is represented by 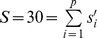. As the shelters are identical in our case, the formula becomes 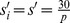.

In Eq. 3, the term *μ*_i_ represents the maximal kinetic constant of entering the shelter and when *xi* = *s’*, the individual rate of entering is null. Each cockroach in shelter *i* has a rate *L_i_* of leaving it. The parameter *θ_i_* is the maximal rate of leaving a shelter; the parameters *p*, *k*, and *n* are adjustment parameters that take into account the inter-individual influence. Greater the population under the shelter *i*, lower is the rate per individual of leaving this shelter.

We performed individual-based stochastic simulations of the model in which the random aspect of the process is automatically incorporated. The simulations were based on the mechanisms defined in the differential system of equations (Eqs.1, 3, and 4). The different steps can be summarized as follows: (1) initial conditions - the number of individuals under each shelter was fixed at 0 and the number outside at 16 and (2) decision process - the position of each individual is checked at each of the time steps *t* (28800 s). The probability of leaving (entering) shelter *i* is given by *L_i_ (E_i_)* (Eqs. 3 and 4). The change of state at time t depends on the comparison between the calculated value *L_i_ (E_i_)* and a random number sampled from a distribution between 0 and 1; if the value is less than or equal to *L_i_ (E_i_)*, the individual leaves (enters) shelter *i*. The probabilities *L_i_* and *E_i_* are updated at each step of the simulation in relation to the number of individuals *x_i_* already present at site *i*. The simulations were performed 900 times, and the distributions of the number of individuals present in shelter *i* in relation to time were calculated.

For each fragmentation level tested, we do not observe any significant difference between the experimental and theoretical fraction of the total population under the shelters (Figure 2, Kruskal-Wallis test with Dunn’s Multiple Comparisons Post-Hoc: observed vs theoretical data. *P*>0.05 for F1, F2, F6, and F10). The concordance between the experimental and theoretical results suggests that the same decision rules are applied, regardless of the fragmentation level.

Based on the experimental and theoretical data, we observe a decrease in the fraction of the total population under the shelters with the fragmentation level (Figure 2, Kruskal-Wallis test with Dunn’s Multiple Comparisons Post-Hoc: F1 vs F2 vs F6 vs F10. For experimental data: *P*<0.05 for all comparisons, except for F1 vs F2. For theoretical data: *P*<0.05 for all comparisons).

### Distribution Among Shelters

**Figure 3:**
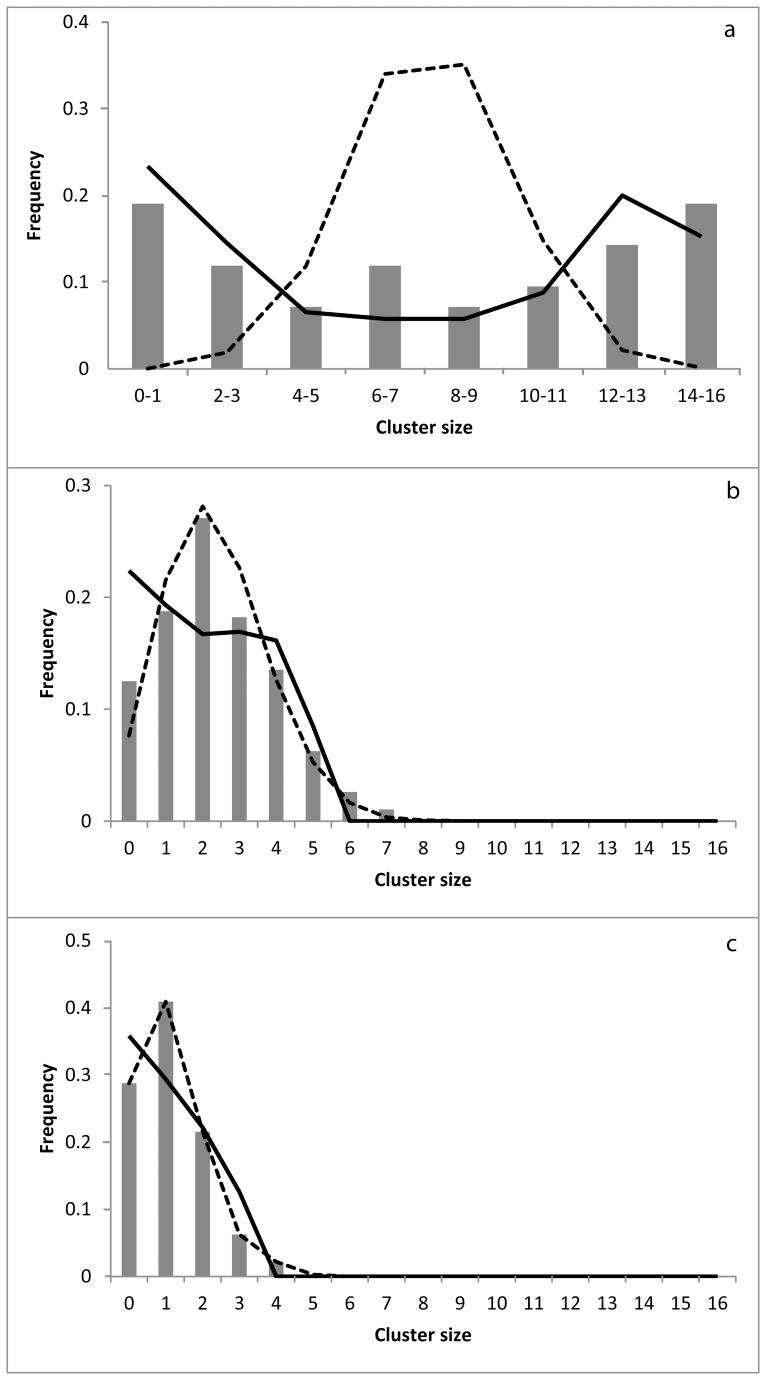
Theoretical and experimental spatial distribution. Frequency distribution of the cluster size at t = 480 minutes for (a) F2, (b) F6, and (c) F10. Histogram: experimental distribution. Solid line: theoretical distribution generated by our model (eq.1). Dashed line: binomial distribution with random behavior. Parameters values: μ = 0.0007, θ = 0.0049, n = 2.5, k = 4, ρ = 1296.

With regard to F1, 95% ±0.08 of the cockroaches aggregate inside the unique shelter because there was no alternative. For F2, F6, and F10, a multimodal distribution of individuals among the shelters highlights an underlying social aggregation process, while a binomial distribution is due to a random process without any interactions between individuals. To test these two hypotheses, consensus or not, we compared the observed cluster size distribution at t = 480 minutes with that obtained from a random process (Figure 3a,b,c). This binomial distribution is built by distributing, with an equal probability, the individuals among *i* shelters (hypothesis of no influence between individuals). The total number of individuals is the number observed in the shelters at t = 480 min (for each fragmentation level: the number of replicates *100). For F6 and F10 (Figure 3b,c), we found a good agreement between the two distributions, suggesting that the experimental distribution of the individuals among the shelters is equivalent to a random one (correlation between the observed and random distribution. F6: Spearman *r* = 0.98, *n* = 9, *P*<0.0001. F10: Spearman *r* = 0.99, *n* = 7, *P*<0.001). For F2 (Figure 3a), the distribution of individuals in the 2 shelters is negatively correlated with a random distribution (correlation between the observed and random distribution. Spearman *r* = −0.80, *n* = 8, P<0.05). These results highlight a strong divergence between these two distributions.

Based on our model, including interactions between individuals (Eq. 1) and with the same method and the same parameters values previously described, we built a theoretical distribution of individuals among the F2, F6, and F10 shelters and obtained a good agreement between the experimental and theoretical datasets (F2: Spearman *r* = 0.80, *n* = 8, *P*<0.05. F6: Spearman *r* = 0.76, *n* = 9, *P*<0.05. F10: Spearman *r* = 0.95, *n* = 7, *P*<0.01).

## Discussion

Considering the ability of most of the cockroach species to aggregate under shelters [22], [51],[52], we sought to assess the response of a group to habitat fragmentation and a consequent increase in the number of resting sites. To this end, we subjected a group of cockroaches to different fragmentation levels without habitat loss, with the total shelter area remaining constant: the number of shelters increases while the carrying capacity of each decreases.

Our experiments with different fragmentation levels provided extensive information on the influence of fragmentation and on the behavioral mechanisms involved in the spatio-temporal dynamics of fragment occupation. Indeed, our results showed that the joint action of the multiplication of shelters and the reduction of their respective area induces a decrease in the fraction of the total sheltered population. Regardless of the duration of the experiment, the total occupancy of the shelters decreases when the number of available shelter increases from 1 to 10.

Moreover, increasing the level of fragmentation entails a transition in the cockroaches’ spatial distribution from an aggregation under one shelter to an equal distribution among them. Faced with two shelters, each with a capacity greater than the size of the population, cockroaches collectively select a single shelter. When six or ten shelters are available, there is a homogeneous distribution, i.e., the sheltered population is equally distributed among the shelters, and a lower total sheltered population is observed.

The experimental and theoretical results of our model converge, showing that the different observed patterns, aggregation or dispersion, can be explained by the same behavioral mechanisms. Aggregation and dispersion are macroscopic expressions of the same rules at the individual level (the same model and parameter values). Irrespective of the level of fragmentation, the probability of being in a shelter results from the combination of individual probabilities to enter and leave the shelter. Consequently, the higher the ratio of the probability of entry/exit is, the greater is the probability of presence. Under our conditions and without taking into account any social influence, cockroach will be more present in large shelters (in our experiments, low fragmentation) than in small ones. Conversely, the individual probability of entering a shelter (*μ*) remains the same, regardless of its surface area (Terramorsi, personal communication).

However, in the presence of congeners, the cockroach residence time under a shelter and its probability of joining it do not only depend on the size (surface) of the fragment. Indeed, the probability of presence involves positive and negative feedback loops based on physical constraints and social interactions [21], [22], [46]. They induce a modulation of the individual probability of leaving the shelter and an amplification process (positive feedback): the probability to stay in the shelter increases with the size of the aggregate [1], [20], [25]. It is noteworthy that inter-individual communication in cockroach species is essentially based on chemical compounds, such as cuticular hydrocarbons [51], [53]–[56]; consequently, the influence of peers on the probability of leaving (retention phenomenon) operates at a very close range.

The size of a shelter also imposes a limit to the number of sheltered individuals: the smaller the shelter is, the fewer individuals it can contain; thus, the probability of joining a shelter decreases with the number of sheltered individuals, leading to a negative feedback loop. Moreover, as the probability of leaving a shelter depends on the number of congeners, an increased fragmentation rate may induce an increased likelihood of leaving the shelter. The coupling of these two non-linear processes involves the existence of a threshold corresponding to a critical number of individuals settled under a shelter [45]. From this threshold value, the number of individuals in the shelters can increase to contain the majority of the population.

The existence of such a threshold allows the association of our model with models of the ideal free distribution (IFD) [44], a theory that assumes that individuals compete for resources, i.e., shelters in our case. According to IFD theory, individuals are distributed evenly over all available sites, independently of the level of fragmentation. This equal distribution of individuals results in the propensity to select the site in which the number of competitors is lowest.

In our study, we showed that, when the shelter size enables cockroaches to reach the threshold value, the set of probabilities to enter/exit modulated by social interactions leads to the emergence of site selection. This binary choice experiment is in agreement with the results of previous studies [21], [22], [24], [45], [47], [56], whereas IFD theory is not able to predict such selection. In fact, through theoretical results obtained with a numerical simulation of cockroaches, we demonstrated that the collective choice of a shelter cannot be obtained via the simple summation of individual responses to identical environmental heterogeneity and without inter-attraction among congeners.

For a small number of shelters (<4), a previous model [20], [57] predicts that cockroaches tend to occupy the minimum number of shelters. They show that with strong crowding effect, corresponding to a small ratio between the carrying capacity of each shelter and the total population, cockroaches are distributed equally in the several shelters. When crowding is weaker or missing, cockroaches tend to aggregate and consequently to occupy a minimum number of shelters. Our experimental results are in agreement with these predictions as the concomitant increase of the fragmentation level and of the crowding lead to a switch from a aggregation pattern for 1 and 2 shelters to a scattering of individuals among the 6 or 10 shelters.

The differences observed between the distributions of individuals confronted with a choice between 2 shelters or 6 and 10 shelters can be explained by the need to reach such a threshold. Indeed, for the 2-shelters scenario, the number of cockroaches will exceed this threshold and trigger the amplification process; in contrast, the dispersion observed under the conditions “6 shelters” and “10 shelters” is explained by a number of cockroaches per shelter generally below this threshold. In both situations of high fragmentation (F6 and F10), the importance of social interactions on the probability of leaving is greatly reduced (see Eq. 1) and is not sufficient to trigger a dynamic of the selection of shelters. In these cases, the cockroach distribution is similar to that predicted by IFD theory.

Through simulations, we are able to confirm that, in the presence of six or ten small shelters, the distributions were similar to those expected if the individuals were non-social.

In our situation, homogeneous distributions result from a simple reduction of the influence of social interactions, without a qualitative modification of the behavior of the cockroaches. In other words, the transition between aggregation and dispersion is simply based on the modulation of a single behavioral process based on interactions, a situation that does not require the use of other types of agonistic behaviors, though such behaviors cannot be completely excluded.

The aggregation of group-living organisms in a patchy environment is a widespread phenomenon observed at different scales for terrestrial and marine organisms, for invertebrates and vertebrates, and for very different purposes [2], [6], [58], [59]. For such species, the spatio-temporal distribution of the population among patches results from the interplay between social amplification and individual responses to environmental heterogeneity. Many of the behaviors described in the literature and leading to amplification [60] strongly indicate that collective patterns and their plasticity reported here are not specific to cockroaches and do not depend on the nature of cues/signal used by the animals. Therefore, these behaviors must be shared with many species. Although many human activities lead to an increase in the patch number associated with a decrease in habitat lost, this is not always the case. In the ocean, fish (e.g., tuna) aggregate around different types of floating objects (e.g. logs, coconut). Fishermen use this behavior for fishing purpose by deploying a large number of artificial floating objects which lead to an increase in the number of patches. Theoretical analysis predicts results similar to our experiments, whereby an increasing number of patches causes a shift from a heterogeneous distribution (selection of a small number of patches) to a homogeneous distribution [61]. Interestingly, theoretical approaches based on game theory and focusing on the ultimate causes of decision making-process predict other collective patterns. For cockroaches, benefits of the aggregation under shelters result from the combination of the protective effect of the shelter and the aggregation which is a cooperation reducing notably the physical stresses [11]. In such situation, the sheltering patterns that we observe may be assumed to be different outcomes of a public game. In this game, insects act as co-operators in an environment constituted by small number of large shelters. A consensus for the selection of a shelter by many conspecifics could emerge from interaction between actors. If the size of the shelters is small, the synergetic effects are weak and insects are homogeneously distributed among many small shelters. In this case, individuals act as free riders and they adopt one of the shelters for its own characteristics. This is in agreement with the prediction of the public good game model of Wang et al. [62], [63]. Interestingly, their model predicts an increase of the cooperation when the population density decreases. Therefore, it would be instructive to conduct further experiments with different cockroach densities to validate this prediction.

We hypothesize that our results are generic properties of aggregation in patchy environments, and our experimental and theoretical methodology can be applied to many species to validate this hypothesis.
